# Comparative Genomic Analysis of Coxsackievirus A6 Strains of Different Clinical Disease Entities

**DOI:** 10.1371/journal.pone.0052432

**Published:** 2012-12-26

**Authors:** Yi-Jen Chen, Shih-Cheng Chang, Kuo-Chien Tsao, Shin-Ru Shih, Shu-Li Yang, Tzou-Yien Lin, Yhu-Chering Huang

**Affiliations:** 1 Department of Pediatrics, Chang Gung Memorial Hospital, Taoyuan, Taiwan; 2 Research Center for Emerging Viral Infections, College of Medicine, Chang Gung University, Taoyuan, Taiwan; 3 Department of Medical Biotechnology and Laboratory Science, College of Medicine, Chang Gung University, Taoyuan, Taiwan; 4 Department of Laboratory Medicine, Chang Gung Memorial Hospital, Taoyuan, Taiwan; University of Ottawa, Canada

## Abstract

**Background:**

Studies regarding coxsackievirus A6 (CVA6) infection were limited. In Taiwan, outbreaks of CVA6 occurred in 2009 and 2010, respectively, but the clinical manifestations were markedly different. We conducted a study to compare the clinical features and genomic sequence between the two years.

**Methodology/Principal Findings:**

In 2009 and 2010, 205 patients with coxsackievirus A6 (CVA6) infection were treated at Chang Gung Memorial Hospital. Detailed clinical features were obtained from 126 inpatients, 62 in 2009 and 64 in 2010. Between the inpatients in 2009 and 2010, no statistically significant difference was noted in terms of demographics, length of hospital stay and laboratory data. Significantly more patients in 2009 presented with herpangina (82%) while more patients in 2010 presented with hand-foot-mouth disease (HFMD; 67%) and skin rash beyond the typical sites for HFMD. Complete genomic sequences were determined and compared for three isolates from patients with herpangina in 2009 and three isolates from patients with HFMD in 2010. The complete sequences showed that 2009 and 2010 CVA6 isolates were indistinguishable by partial VP1 genes, but there were 5 unique nucleotide changes in 3′ UTR, and 23 out of 2201 (1%) amino acids were different. 2010 viruses underwent the largest number of amino acid changes in 3CD protein, which is the precursor of both 3C protease and 3D polymerase.

**Conclusions:**

Since 2008 in Finland, outbreaks of HFMD due to CVA6 were noted internationally. CVA6 of different genetic background may cause different clinical manifestations such as herpangina and HFMD.

## Introduction

Enterovirus infections resulted in a variety of disease, ranging from asymptomatic infection, herpangina, hand-foot-month disease (HFMD), to more severe diseases such as menigoencephalitis, myocarditis, and neonatal sepsis [1]. Group A coxsackieviruses (A1-10, 16, 22) are the most common viruses in herpangina patients. Coxsackievirus A16 and enterovirus 71 (EV71) are most frequently implicated in HFMD, and other serotypes, including coxsackieviruses A4-10, A24, coxsackieviruses B2-5, and echovirus 18, can also cause HFMD [2].

Studies regarding coxsackievirus A6 (CVA6) infection were limited [3]–[10]. In 2008, an outbreak of HFMD with onychomadesis caused by CVA6 was reported in Finland [3], [4]. Then CVA6 was recognized as an emerging cause of epidemic HFMD, and thereafter several outbreaks of HFMD caused by CVA6 were reported from Singapore in 2008 [5], Taiwan in 2010 [9], Japan in 2011 [10], and the United States in 2012 [11].

After EV71 epidemic in 1998, virus surveillance for enterovirus activity has been monitored by the Centers for Diseases Control (CDC) of Taiwan. CVA6 ranked among the top five serotypes in Taiwan between 2001 and 2010. In 2009, CVA6 was the most common endemic enterovirus, and it ranked the 3^rd^ in 2010 [12]. In our previous study [8] focusing on CVA6 infection from 2004 to 2009, 76.6% of the patients presented as herpangina, and HFMD accounted only for 12.8%. However, in 2010 we found most children infected with CVA6 presented with oral ulcers, various vesicular skin lesions and even onychomadesis, a picture totally different from what we saw previously. These observations prompted us to conduct a study to figure out the differences from clinical manifestations of the patients with CVA6 infection to complete sequence analysis of CVA6 clinical isolates identified between the years 2009 and 2010.

## Materials and Methods

### Ethic Statement

The study was approved by the Institutional Review Board of Chang Gung Memorial Hospital. Since the data obtained in this study were collected from the patients, who just received regular medical management, by retrospective medical charts review, a written consent from the patients was waived.

### Patients

A total of 329 patients with culture-proven coxsackievirus A6 (CVA6) were identified from the virological logbook of Chang Gung Memorial Hospital, 151 patients in 2009 and 178 in 2010. The proportion of CVA6 among total enterovirus isolates was 22.2% in 2009 and 20.5% in 2010. Excluding 124 patients without complete demographic data, mostly from outside institutes, 205 patients were included. Of these 205 patients, 62 and 64 were inpatients in 2009 and 2010, respectively. From the 79 outpatients, only demographic data were obtained. The detailed clinical features were obtained from the 126 inpatients.

Medical charts of the 126 inpatients were reviewed retrospectively. The demographics, underlying disease, hospitalization duration, clinical manifestations, laboratory data, final diagnosis, clinical outcome, and complications were collected.

### Definitions

Herpangina was defined as well-characterized vesicular enanthem and then ulcers of the fauces and soft palate with presentation of fever, sore throat, and decreased appetite. HFMD also had oral ulcers but chiefly on the buccal mucosa and tongue, accompanied with typical vesicular rashes most commonly on the extensor surfaces of the hands, feet, knees and buttocks. Leukocytosis was defined as WBC count ≥17.5×10^3^/µL. Viral co-infection was defined as virus other than enterovirus was also detected in viral isolation.

### Virus Culture

All viral cultures were done via throat swab and the details of the method were described previously [8], [13]. Briefly, specimens were inoculated into human embryonic fibroblast (MRC-5), MDCK, HEp-2 and RD cell cultures. When enteroviral cytopathic effect involves more than 50% of the cell monolayer, indirect fluorescent antibody staining with panenteroviral antibody was done to identify enterovirus. Antibodies against enterovirus (pan-enterovirus, group specific and type-specific antibodies) were added to identify serotype-specific enteroviruses. The monoclonal antibodies, covering 18 serotypes (Poliovirus 1–3; coxsackievirus A9, A16, A24; coxsackievirus B1–6; echovirus 4, 6, 9, 11, 30; EV71), were from a commercial kit (Chemicon International, Temecula, CA, USA), and monoclonal antibodies against coxsackievirus A2, A4, A5, A6 and A10 were provided by CDC–Taiwan since 2006. [14] Some selected isolates were sent to the Centers for Diseases Control of Taiwan for confirmation/determination of the serotypes by a reverse transcription-seminested polymeras chain reaction assay [15].

### Complete Genome Sequencing of Coxsackievirus A6

Three isolates from patients diagnosed as herpangina in 2009, and three isolates from HFMD patients in 2010 were selected for complete sequencing analysis. Viral RNA was extracted from virus culture using the Viral RNA Extraction Miniprep System Kit (Viogene, Sunnyvale, CA, U.S.A.) according to the protocol recommended by the manufacturer. The cDNA synthesis was performed using RT-primer and M-MLV reverse transcriptase (ReverTra Ace; Toyobo, Osaka, Japan). Because large genome difference was observed between prototype and recent field isolates and no other complete genome was available in GenBank at the time of this study, the whole genome sequencing of CVA6 was determined by PCR-based primer walking using the primers listed in Table S1. The sequences obtained in this study were deposited in GenBank under the accession numbers JQ946050–JQ946055.

### Sequence Analysis

Multiple sequence alignment was conducted using the ClustalW multiple alignment program within the BioEdit Sequence Alignment Editor package, version 7.0.9.0. [16]. The coding region sequences were translated to amino acid sequences and the percent identities between pairs of sequences were calculated by BioEdit. The unique nucleotide and amino acid substitutions of CVA6 isolates in 2010 were analyzed by comparing with the CVA6 genomes in 2009. The sequence data of the prototype strain Gdula were also used for comparison and analysis. The phylogenetic tree based on VP1 genes was performed using the neighbor-joining method in the MEGA tree program, with a bootstrap value of 1,000 [17].

### Statistical Analysis

For categorical variable, we used chi-square test, and the Mann–Whitney *U* test was used for continuous variables. P values are two-sided, and they are considered significant if ≤0.05. All analysis were performed with the software SPSS, version 17.0.

## Results

### Clinical Aspect

**Figure 1:**
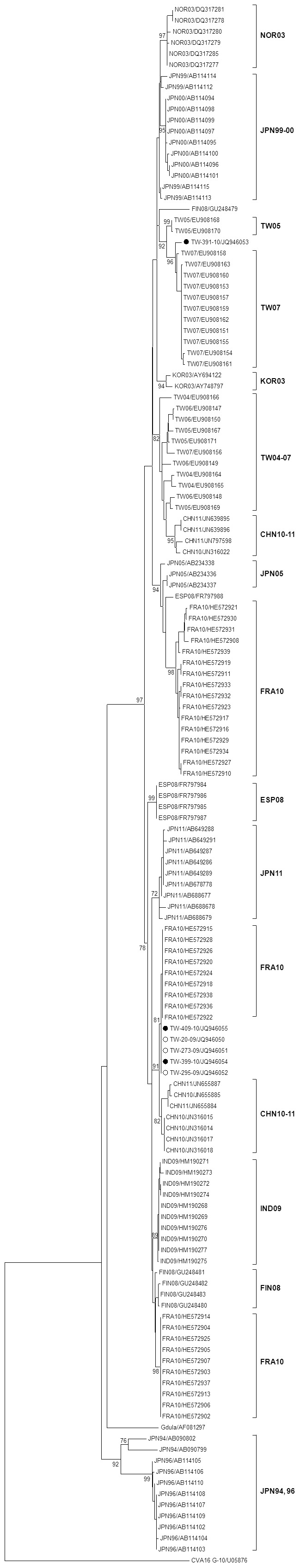
Monthly Distribution of Cox-A6 infection children in 2009 and 2010.

Of the 205 patients included, 99 patients were identified in 2009 and 106 patients in 2010. The median age was 2.4 and 2.6 years for children in 2009 and 2010, respectively; 68% and 55% of the patients in 2009 and 2010, respectively, were less than three years of age. The male-to-female ratio was 1.41 and 1.79, respectively. There was no statistically significant difference for age and gender between the two groups. Figure 1 showed the monthly distribution of these children, with a peak in June and August, respectively.

Table 1 illustrates the demographics, clinical manifestations and laboratory data of the 62 and 64 hospitalized patients in 2009 and 2010, respectively. Demographics of the patients as well as the length of hospital stay were comparable for both groups. Leukocytosis and elevated serum C-reactive protein (CRP) level >40 mg/L were noted in a substantial proportion of the patients in both groups but there was no statistically significant difference between the two groups.

**Table 1:** Comparisons of demographics, clinical manifestations and laboratory data between inpatients with coxsackievirus A6 infection in 2009 and 2010 in Taiwan.

Characteristics	2009 (n = 62) No. (%)	2010 (n = 64) No. (%)	*p* value
**Age (year) (mean**±SD)	2.40±1.58	2.85±2.12	0.246
<3 years, n(%)	43 (69.4)	40 (62.5)	0.417
**Male gender, n (%)**	35 (56.5)	41 (64.1)	0.383
**Underlying disease, n (%)**	9 (14.5)	12 (18.8)	0.524
**Length of hospitalization (day)**			
Mean±SD	4.5±1.44	4.86±1.90	
Median (range)	4 (3–11)	4 (2–13)	0.529
**Fever**	62 (100)	63 (98.4)	0.323
Duration (mean±SD, days)	2.66±0.99	2.52±1.29	
Fever ≥3 days	29 (46.8 )	29 (45.3 )	0.869
Fever ≥39°C	49 (79)	42 (65.6)	0.099
**Oral ulcer**	58 (93.5)	56 (87.5)	0.248
Lesion site			0.001
Soft palate only	52 (90)	32 (57)	<0.001
Beyond soft palate	5(9.6)	16(28.6)	
Soft palate spared	1(1.9)	7(12.5)	
**Skin rash**	8 (12.9)	47 (73.4)	<0.001
Typical sites for HFMD	5 (62.5)	27 (57.4)	0.789
Trunk ± neck	3 (37.5)	13 (27.7)	0.571
Face	0 (0)	10 (21.3)	0.149
Perioral	0 (0)	5 (10.6)	0.333
Myoclonic jerk	19 (30.6)	23 (35.9)	0.529
Febrile Seizure	2 (3.2)	1 (1.6)	0.54
**Clinical Diagnosis**			<0.001
HFMD	6 (9.7)	43 (67.2)	
Herpangina	51 (82.3)	12 (18.8)	
Pharyngitis/tonsillitis	3 (4.8)	4 (6.2)	
Croup	1(1.6)	1(1.6)	
others	1(1.6)	4 (6.2)	
Peak leukocyte count (x1000/uL)	15.10±4.63	15.46±5.51	0.93
leukocyte count >17500/uL	17 (27.4)	21 (32.8)	0.509
peak CRP (mg/L)	43.09±40.16	42.53±36.19	0.741
CRP>40	27 (43.5)	25 (39.1)	0.834
Other viral co-infection*	7 (11.3)	5 (7.8)	0.506

HFMD, hand-foot and mouth disease; CRP, C-reactive protein.

* Excluding the patients with co-infection, the difference of clinical manifestations was still significant statistically.

Nearly all the patients had fever, with a median duration of 2 days and ranging from 1 to 5 days for patients in 2009 and 0 to 7 days in 2010. Although around 90% of the patients in each year had oral ulcers, 90% of the patients in 2009 had oral ulcers limited to posterior pharyngeal wall and soft palate only, while 43% of the patients in 2010 had ulcers (P<0.001) beyond posterior pharyngeal wall and soft palate. Likewise, the presentation of skin rash and the distribution of clinical diagnoses were significantly different between the patients in two groups (P<0.001 for both). 82% of the patients in 2009 had a clinical diagnosis of herpangina, while 67% of the patients in 2010 presented with HFMD. Moreover, 44% of the patients in 2010 had skin rashes beyond the typical sites for HFMD, including trunk, neck, face, and perioral area. All the patients in these two years recovered uneventfully.

There were seven and five inpatients with other viral co-infection in 2009 and 2010, respectively. The difference of clinical manifestations described above was still significant after excluding the patients with co-infection.

### Complete Genome Sequencing of Coxsackievirus A6

During the study period, the genome of prototype strain Gdula was the only sequence available for CVA6 complete genome analysis. By comparison with the prototype strain, the recent CVA6 isolates in 2009–10 had the nucleotide sequence identities of approximately 88% in 5′ UTR, and 82–83% in P1 region. However, the sequence identities of 2009–10 viruses were less than 80% in both P2 and P3 regions (Table S2). Recombination between enteroviruses of various serotypes may explain the findings. However, when comparing the strains between 2009 and 2010, we found no obvious difference in similarity and bootscan analysis. Therefore, consensus degenerate primers designed from one prototype strain *were limited and* the complete genome in this study was sequenced largely by primer-walking strategy, especially for P2 and P3 regions.

**Figure 2:**
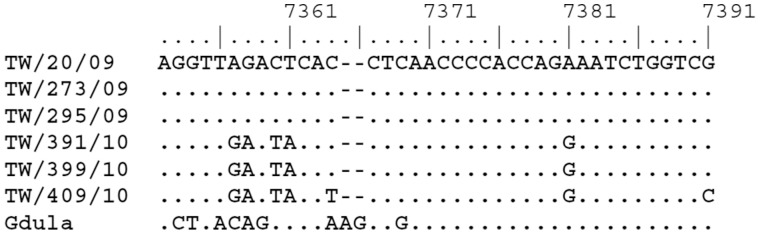
Phylogenetic analysis of CVA6 VP1 genes. Partial VP1 sequences (nt 2957 to 3306 according to Gdula numbering) of 6 CVA6 strains obtained from this study and 135 partial VP1 sequences of reference strains derived from GenBank were used to perform phylogenetic analysis. The phylogenetic tree was constructed using the neighbor-joining method with 1,000 bootstrap replications, as implemented in MEGA version 4. CVA16/G-10 (Accession No. U05876) was used as an outgroup. Bootstrap values over 70% are shown at the branch nodes. GenBank accession numbers are indicated after the slash. Open circle indicated 2009 CVA6 and black circle indicated 2010 CVA6. Abbreviations: CHN, China; ESP, Spain; FIN, Finland; FRA, France; IND, India; JPN, Japan; KOR, Korea; NOR, Norway; TW, Taiwan.

To investigate genetic basis of 2010 CVA6 isolates for the changing in clinical presentations, the phylogenetic tree was constructed based on partial CVA6 VP1 genes. As shown in Figure 2, one of 2010 CVA6 strain (TW/391/10) was closely related to the strains previously reported in Taiwan during 2004–07, in Japan during 1999–2000 and 2005, in Korea in 2003, and in Norway in 2003. Interestingly, a new lineage of CVA6 emerging recently in Taiwan since 2009 was clustered together with Finland and Spain strains in 2008, and Japan strains in 2011, which were associated with outbreaks of HFMD with onchomadesis [3], [4], [7], [10]. This new lineage was also similar to recent isolates in China, France and Indian [18], [19]. However, the phylogenetic results showed that 2009 and 2010 CVA6 isolates were indistinguishable by partial VP1 genes, although CVA6 infections between these two years were easily discernible from clinical presentations.

**Figure 3:**
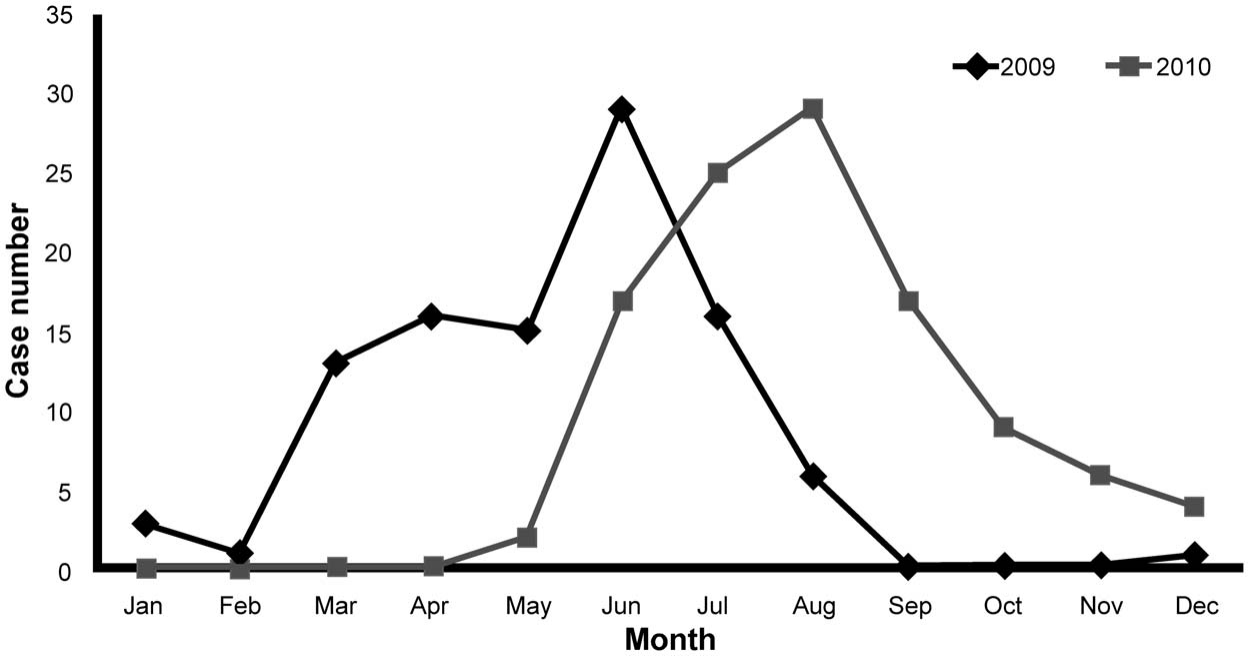
Alignment of the nucleotide sequences of 3′ UTR of CVA6. The first three strains (Accession Nos. JQ946050–JQ946052) were isolated from HFMD patients with small skin rush in 2009, but other three strains (Accession Nos. JQ946053–JQ946053) were isolated from HFMD patients with onchomadesis in 2010. The ‘-‘ denotes a gap and the ‘.’ denotes sequence identity in the sequence. Numbering is based on coxsackievirus A6 strain Gdula (accession No. AY421764).

To identify the genomic sequences of CVA6 associated with distinct phenotype, three complete genomes of CVA6 from each year were compared with each other. The untranslated regions (UTR) present at both end of CVA6 genome are crucial for translation and replication of viral genome. There were 5 unique nucleotide changes in 3′ UTR of 2010 viruses, but no consistent nucleotide change was observed in 5′ UTR (Fig. 3). Comparison of complete genomes showed that 23 out of 2201 (1%) amino acids consistently differentiated the 2010 from the 2009 viruses (Table 2). The viral capsid proteins (VP1-VP4) are known to involve in receptor binding and antigenic property, thus having a role in eliciting immune response. The capsid protein sequences of 2010 viruses differed from 2009 virus at only 1 and 2 amino acids in VP2 and VP3 protein, respectively. Among two viral encoded proteases (2A and 3C proteases) that are responsible for the process of viral polyprotein, 2010 viruses had 4 and 3 consistent amino acid changes in 2A and 3C proteases, respectively. The viral RNA-dependent RNA polymerase (RdRp), denoted 3D polymerase, is required for viral genome replication. 3D polymerase had 13 amino acid changes, which was composed mostly of amino acid changes among mature viral proteins. 3CD protein, which is the precursor of both 3C protease and 3D polymerase, has the protease activity of 3C protein domain but no RdRp activity. 2010 viruses underwent the largest number of amino acid changes in 3CD protein. Besides, low nucleotide (88.3±0.23%) and amino acid (97.7±0.05%) identities in P3 region between 2010 and 2009 viruses agreed with high rate of mutation occurred in the corresponding sequences (Table 3). Notably, three clusters of amino acid changes were identified in 2010 viruses. Two clusters harboring two amino acid changes (underlined), ‘KGH’ and ‘IL’, were located at positions 101–103 in 2A, and 56–57 in 3C, respectively. The other cluster included 4 amino acid changes (underlined), ‘IDKIKK’, was located at positions 165–170 in 3D. Overall, the results suggest that unique nucleotide and amino acid sequences of 2010 viruses may be associated with the important, but as yet unknown, virus functions that lead to altered pathogenesis of CVA6.

**Table 2:** The unique amino acid changes of coxsackievirus A6 in 2010.

Gene		2009			2010		
	Gdula	20	273	295	391	399	409
VP2							
236	I	V	V	V	I	I	I
VP3							
49	I	V	V	V	I	I	I
65	N	N	N	N	S	S	S
2A							
26	N	T	T	T	N	N	N
57	N	N	N	N	D	D	D
101	V	V	V	V	K	K	K
103	H	Y	Y	Y	H	H	H
3C							
56	V	V	V	V	I	I	I
57	L	V	V	V	L	L	L
181	S	T	T	T	S	S	S
3D							
8	P	P	P	P	S	S	S
33	V	V	V	V	I	I	I
75	T	R	R	R	K	K	K
139	S	N	N	N	S	S	S
165	L	L	L	L	I	I	I
168	I	V	V	V	I	I	I
169	K	R	R	R	K	K	K
170	K	R	R	R	K	K	K
190	T	T	T	T	A	A	A
260	E	E	E	E	N	N	N
308	T	A	A	A	S	S	S
342	L	L	L	L	S	S	S
436	T	T	T	T	A	A	A

**Table 3:** Pairwise nucleotide and amino acid identities of coxsackievirus A6 isolated between 2009 and 2010.

Gene	Sequence comparison between 2009 and 2010 strain			
	Nucleotide		Amino acid	
	Identity (%)	Average (%)	Identity (%)	Average (%)
5′ UTR	93.7–99.6	96.9±2.35	–	–
P1 region	93.9–97.4	95.7±1.41	99–99.4	99.2±0.17
VP4	88.9–99.5	96.2±5.18	97.1–100	98.1±1.45
VP2	91.7–98.4	94.9±2.60	98.8–99.2	99.1±0.27
VP3	90.9–91.2	91.1±0.15	98.1–98.1	98.1±0
VP1	93.7–99.7	97.2±2.40	99.7–100	99.6±0.41
P2 region	95.7–96	95.9±0.12	98.8–99.1	99.0±0.13
2A	89.3–90.2	89.8±0.39	96–96.7	96.5±0.35
2B	92.3–92.9	92.6±0.21	99–100	99.3±0.50
2C	99.6–99.8	99.7±0.07	100–100	100±0
P3 region	87.5–88.9	88.3±0.23	97.6–97.7	97.7±0.05
3A	99.2–100	99.6±0.28	98.8–100	99.2±0.60
3B	97–100	98.2±1.25	95.5–100	98.5±2.25
3C	84.2–86.2	85.5±0.92	98.4–98.4	98.4±0
3D	86.7–86.9	86.8±0.07	97–97.2	97.1±0.10
3′ UTR	83.3–88.1	86.5±2.40	–	–

## Discussion

From the present study we found clinical manifestations of the patients infected with CVA6 were different markedly between the year 2009 and 2010. Most patients in 2009 presented with herpangina. While the patients in 2010, oral ulcers were frequently seen in the sites other than soft palate and pharyngeal wall, which are commonly seen in herpangina, and three-fourths of the patients had additional skin rashes over trunk, neck and face, which are not typical sites for HFMD. These findings were also observed in another hospital in Taiwan, 2010 [9]. They reported that 22% of patients with CVA6 infection had eruptions around the perioral area, 30% had rashes over trunk ± neck and 6.5% had generalized skin eruptions. Onychomadesis 1–2 months following HFMD were also noted. Onychomadesis were reported to be an characteristic feature for CVA6 infection in Finland in 2008 [3], [4], and later in Japan in 2011 [10]. However, in the present study we could not find the description of onychomadesis from the charts review and could not know the exact incidence rate of onychomadesis.

In 2009, 82.3% of patients were diagnosed as herpangina; only nearly 10% of the patients were HFMD, which were consistent with our previous study [8]. In contrast, most patients in 2010 were diagnosed as HFMD. Data from Taiwan CDC revealed that CV A2, A4, A5, A6, and A10 were the most common serotypes implicated in herpangina from 2000 to 2005 [20]. Herpangina outbreak related to CVA6 was also noted in Japan in 2005 [21]. In contrast, CVA6 has been found to be an emerging causative agent for HFMD outbreaks in Finland [4] and Singapore [5] since 2008 and also in India in 2009 [18]. After the outbreak of HFMD in Taiwan in 2010, the outbreaks were also from Japan in 2011 [10] and from the United States in 2012 [11].

Generally, patients with HFMD have a temperature of 38°C to 39°C lasting 1 to 2 days [2]. In the present study, almost all the patients in 2010 had fever, and the fever lasted longer (45% of cases ≥3 days) and higher (two-thirds ≥39°C). In addition, a substantial proportion of the patients had leukocytosis and elevated serum CRP>40 mg/L. All these findings indicated a higher disease severity caused by the CVA6 strain of 2010.

The VP1 gene of enterovirus genome has been extensively used in phylogenetic analysis due to high degree of diversity among virus serotypes [22]. However, in the present study, the entire genome complexity was greater than anticipated based on partial VP1 sequences, which may misinterpret the phylogenetic relationship. Furthermore, the genome sequence of the prototype strain Gdula isolated in 1949 is obviously different from current strains in this study (Tables S2 and S3) [23] Therefore, the complete genome sequencing of current CVA6 and other serotype enteroviruses may contribute to the study of enterovirus evolution and genetic variations.

The capsid protein region of 2010 CVA6 possessed 3 unique amino acid substitutions; however, preliminary data from indirect immunofluorescent assay (IFA) suggested that these mutations are not sufficient to produce obvious antigenic changes when compared with 2009 viruses (data not shown). Most mutations reported in this study occurred in non-structural proteins and their functional domains have been defined in recent years. For example, the residues His-20, Asp-38, and Cys-109 comprising catalytic triad of poliovirus 2A protease are essential for inhibition of cap-dependent translation of host mRNA by cleavage of eukaryotic initiation factor 4G (eIF4G) [24], [25]. Poliovirus 3C protease contains a His-40, Glu-71, Cys-147 catalytic triad and a conserved KFRDIR sequence for RNA recognition [26], [27]. However, none of the mutations in this study were found among these functional sites (Table 2). Following the ‘right hand’ structure of 3D polymerase, two mutations of 3D polymerase at positions 8 and 33, and one at position 436 were located in the ‘index’ and ‘thumb’ sub-domain, respectively. The ‘ring’ sub-domain contained the clustered mutation ‘IDKIKK’. Besides, mutations at positions 308 in motif B and 342 in motif D were found in the ‘palm’ sub-domain [28], [29]. Extensive interactions between various sub-domains are important for virus polymerase function, including NTP binding, RNA binding and elongation [30]. In addition to coding region, 2010 CVA6 genome also contained 5 unique nucleotide changes in 3′ UTR, in which RNA secondary and tertiary structures existed is important for RNA replication [31]. Nevertheless, 3′ UTR, as well as all structural and non-structural proteins were known to participate in virus lifecycle, the functional effects of mutations in various regions of viral genome need to be investigated further by mutational analysis.

Some limitations should be noted in this study. First, it is a retrospective study. There may be some loss of chart record about the pattern and sites of skin rash. Inter-observer variation may exist, too. This could explain why the proportion of perioral rashes in the present study was lower (only 10.6% of the cases) than that in other studies (22% in one study from Taiwan [9] and 41% (facial rash) in the recent outbreak in the US [11]). Additionally, records of long-term follow-up were lacking, therefore we could not analyze the characteristic manifestations such as the nail abnormalities and desquamation weeks to months after acute episodes. Finally, there were only three isolates representing the 2009 and 2010 epidemics, respectively. More genetic information of 3'-UTR and RdRP from more isolates are needed to confirm the findings in the present study and to define the correlation between genetic background and clinical manifestations.

### Conclusions

Since 2008 in Finland, outbreaks of HFMD due to CVA6 were noted internationally. In addition to herpangina, CVA6 has been an emerging cause of epidemic HFMD other than EV 71 and CVA16. The presentation of HFMD due to CVA6 was somewhat different from that of typical HFMD due to EV 71 and CVA16. Different clinical manifestations caused by CVA6 may result from the different genetic background of viral strains.

## Supporting Information

Table S1 **Sequences of primers used in this study.**
(DOC)

Table S2 **Comparison of nucleotide identities of prototype coxsackievirus A6 strain (Gdula) with circulating strains in 2009–10.**
(DOC)

Table S3 **Comparison of amino acid identities of prototype coxsackievirus A6 strain (Gdula) with circulating strains in 2009–10.**
(DOC)
