# Correction: Comparative Genomic Analysis of Coxsackievirus A6 Strains of Different Clinical Disease Entities

**DOI:** 10.1371/annotation/cc9aa566-b1a7-4dd1-8872-7ecfde4a3f07

**Published:** 2013-06-21

**Authors:** Yi-Jen Chen, Shih-Cheng Chang, Kuo-Chien Tsao, Shin-Ru Shih, Shu-Li Yang, Tzou-Yien Lin, Yhu-Chering Huang

Figure 3 should be figure 1, figure 2 should be figure 3, and figure 1 should be figure 2. The word "rash" is spelled incorrectly in the legend for figure 3. The correct sentence should be: "The first three strains (Accession Nos. JQ946050–JQ946052) were isolated from HFMD patients with small skin rash in 2009, but other three strains (Accession Nos. JQ946053–JQ946053) were isolated from HFMD

patients with onchomadesis in 2010. The ‘-‘ denotes a gap and the ‘.’" 

