# Supplementary material for: Comparative Genomic Analysis of Coxsackievirus A6 Strains of Different Clinical Disease Entities
Source: PLoS One. 2012 Dec 26;7(12):e52432. doi: 10.1371/journal.pone.0052432 (PMC3530459; doi:10.1371/journal.pone.0052432)
Supplement: Table S1 — Sequences of primers used in this study. (DOC) [file pone.0052432.s001.doc]

Table S1. Sequences of primers used in this study

| Primer | Sequence (5’ to 3’) | Location | Region |
| --- | --- | --- | --- |
| FL-F-1C | TTAAAACAGCCTGTGGGTTG | 1-20 | 5’UTR |
| panEV-F | CCCCTGAATGCGGCTAATC | 458-476 | 5’UTR |
| panEV-R | GCTGCTTATGGTGACAATC | 629-610 | 5’UTR |
| 5'UTR-EV-F-FW2 | CCCTCTTAGTGGCTGCAATC | 1314-1333 | VP2 |
| VP1-222-FW2-R | TGGGGCAATAGTTAGTGTGA | 1678-1659 | VP2 |
| 224-F | GCIATGYTIGGIACICAYRT | 2159-2178 | VP3 |
| 2715-R | TTCACCTCCACAACYCCTACYAGC | 2715-2692 | VP1 |
| 222-R | CNCCNGGNGGNAYRWACAT | 2903-2885 | VP1 |
| 3311-F | ATAACTAACACTGCAACCGACC | 3272-3293 | VP1 |
| 3441-R | GCCCARTCATTATGAGTGGC | 3441-3422 | 2A |
| 4069-R | GCMTCCATCYTAGGTATCCCTA | 4098-4077 | 2B |
| 4613-F | GTGGTCACAGTYATGGACGATC | 4613-4634 | 2C |
| 5477-R | GTYGCCGTRCGGAGRATRGGTT | 5406-5385 | 3B |
| 6150-F | GCAGGCRTTGTTCTCYAAGTAT | 6127-6148 | 3D |
| 5332-F | GTTTTCCCAGTCACGACTTYCARGGBGCKTA | 6785-6815 | 3D |
| PCR primer^a^ | AAGCAGTGGTATCAACGCAGAGT |  |  |
| RT-primer^a^ | AAGCAGTGGTATCAACGCAGAGTACT(30)VN |  | 3’UTR |

^a^The sequences are derived from PCR and CDS primer in the SMART PCR cDNA Synthesis Kit (Clontech Laboratories, Inc. CA, USA). Numbering is based on coxsackievirus A6 strain Gdula (accession No. AY421764). Key to degenerate nucleotides: I = Inosine, K = T+G, M = A+C, R = A+G, W = T+A, Y = C+T, B = C+G+T, V = A+G+C, N = A+T+C+G
