# Supplementary material for: Comparative Genomic Analysis of Coxsackievirus A6 Strains of Different Clinical Disease Entities
Source: PLoS One. 2012 Dec 26;7(12):e52432. doi: 10.1371/journal.pone.0052432 (PMC3530459; doi:10.1371/journal.pone.0052432)
Supplement: Table S2 — Comparison of nucleotide identities of prototype coxsackievirus A6 strain (Gdula) with circulating strains in 2009–10. (DOC) [file pone.0052432.s002.doc]

Table S2. Comparison of nucleotide identities of prototype coxsackievirus A6 strain (Gdula) with circulating strains in 2009-10

| Gene | Identity (%) between Gdula strain and | | | | | |
| --- | --- | --- | --- | --- | --- | --- |
|  | 20/09 | 273/09 | 295/09 | 391/10 | 399/10 | 409/10 |
| 5’UTR | 88.1 | 88.2 | 88.2 | 88.1 | 88.4 | 88.0 |
| P1 region | 82.3 | 82.2 | 82.2 | 82.7 | 82.4 | 83.0 |
| VP4 | 79.2 | 79.2 | 79.2 | 80.7 | 78.7 | 81.2 |
| VP2 | 83.3 | 83.7 | 83.7 | 83.7 | 83.2 | 84.1 |
| VP3 | 78.8 | 78.8 | 78.8 | 80.6 | 80.6 | 80.9 |
| VP1 | 83.5 | 82.6 | 82.6 | 83.0 | 83.2 | 83.4 |
| P2 region | 79.8 | 79.8 | 79.8 | 79.8 | 79.7 | 79.9 |
| 2A | 78.9 | 78.9 | 78.9 | 78.9 | 78.9 | 79.3 |
| 2B | 80.1 | 79.8 | 79.8 | 80.1 | 79.5 | 79.8 |
| 2C | 80.1 | 80.1 | 80.1 | 80.1 | 80.1 | 80.1 |
| P3 region | 77.1 | 77.1 | 77.1 | 77.2 | 77.2 | 77.1 |
| 3A | 76.0 | 75.6 | 75.6 | 75.6 | 75.2 | 75.6 |
| 3B | 74.2 | 74.2 | 74.2 | 72.7 | 74.2 | 74.2 |
| 3C | 78.3 | 78.3 | 78.3 | 77.6 | 77.8 | 77.6 |
| 3D | 77.0 | 77.1 | 77.1 | 77.6 | 77.5 | 77.3 |
| 3’UTR | 73.8 | 73.8 | 73.8 | 69.0 | 69.0 | 66.7 |
