# Supplementary material for: Comparative Genomic Analysis of Coxsackievirus A6 Strains of Different Clinical Disease Entities
Source: PLoS One. 2012 Dec 26;7(12):e52432. doi: 10.1371/journal.pone.0052432 (PMC3530459; doi:10.1371/journal.pone.0052432)
Supplement: Table S3 — Comparison of amino acid identities of prototype coxsackievirus A6 strain (Gdula) with circulating strains in 2009–10. (DOC) [file pone.0052432.s003.doc]

Table S3. Comparison of amino acid identities of prototype coxsackievirus A6 strain (Gdula) with circulating strains in 2009-10

| Gene | Identity (%) between Gdula strain and | | | | | |
| --- | --- | --- | --- | --- | --- | --- |
|  | 20/09 | 273/09 | 295/09 | 391/10 | 399/10 | 409/10 |
| P1 region | 95.6 | 95.6 | 95.6 | 95.5 | 95.5 | 95.5 |
| VP4 | 85.5 | 85.5 | 85.5 | 84.1 | 85.5 | 84.1 |
| VP2 | 97.3 | 97.3 | 97.3 | 97.7 | 97.3 | 97.3 |
| VP3 | 97.2 | 97.2 | 97.2 | 97.2 | 97.2 | 97.2 |
| VP1 | 95.7 | 95.4 | 95.4 | 95.1 | 95.4 | 95.7 |
| P2 region | 97.4 | 97.4 | 97.4 | 97.4 | 97.4 | 97.6 |
| 2A | 96.0 | 96.0 | 96.0 | 95.3 | 96.0 | 96.7 |
| 2B | 96.0 | 96.0 | 96.0 | 97.0 | 96.0 | 96.0 |
| 2C | 98.5 | 98.5 | 98.5 | 98.5 | 98.5 | 98.5 |
| P3 region | 93.6 | 93.6 | 93.6 | 93.2 | 93.2 | 93.2 |
| 3A | 90.7 | 90.7 | 90.7 | 90.7 | 89.5 | 90.7 |
| 3B | 90.9 | 90.9 | 90.9 | 90.9 | 86.4 | 90.9 |
| 3C | 94.5 | 94.5 | 94.5 | 95.1 | 95.1 | 95.1 |
| 3D | 93.9 | 93.9 | 93.9 | 93.3 | 93.3 | 93.1 |
